# Antibacterial, antioxidant and anti-inflammatory activity zinc-titanium dioxide nanocomposite

**DOI:** 10.6026/97320630019638

**Published:** 2023-05-31

**Authors:** Mary Sheloni Missier, Mahesh Ramakrishnan, Saravana Dinesh Sudalaimani Paulpandian, Shanmugam Rajeshkumar, Milling Tania

**Affiliations:** 1 Department of Orthodontics, Saveetha Dental College and Hospital, SIMATS, Saveetha University, Chennai, Tamilnadu, India

**Keywords:** Zinc-Titanium nano-composite, lemon extract, antibacterial, antioxidant, anti-inflammatory agent

## Abstract

The current study concentrated on the green synthesis of Zinc-titanium dioxide nano-composite (Zn-TiO2 NC) through the use of lemon extract, optimizing the different experimental factors required for the formation and stability of nanocomposite. The preparation of nanocomposite was confirmed by the observation of the colour change and the surface plasmon resonance band was found at 380 nm, utilizing UV-Visible analysis. The TEM analysis, the morphological features of the prepared nanocomposite was identified to be spherical shape with mean particle size of 25 nm. In addition, the antibacterial, antioxidant, and anti-inflammatory activity of this nano-composite were also investigated. The biosynthesized nanocomposite showed excellent antibacterial activity against S. mitis and S. mutans. The obtained results indicate that the antioxidant and anti-inflammatory activity of this nanocomposite is significant. This bioactive nanocomposite can be used as an effective antibacterial, antioxidant and anti-inflammatory agent in biomedical and pharmacological fields for future applications.

## Background:

Zinc-derived nanoparticles, whether chalcogens (ZnO, ZnS, ZnSe) or metal-non-metal polymers, are increasingly studied worldwide in various applications due to the reduced ability of the charge carrier to rebind energy band structures [1]. Due to their important applications in a variety of fields, control sizes, compositions, orientations, and morphological nanomaterials derived from Zn can be manufactured by physical or chemical means. Among the different conventional approaches(co-water, microwave assisted, hydrothermal, steam transport, precipitation methods, laser ablation), green approaches are the most environmentally friendly, cost-effective and easy-to-scale synthesis methods of nanoparticles [2,3,4].The only nanostructures of zinc, ZnO, ZnS, and ZnSe, have been studied in detail for their excellent properties and applications. ZnO is one of the most promising semiconductor materials with excellent physical and chemical properties. Nanocomposites derived from Zn-doped/coated are lead materials for environmental applications. Surface modification of Zn-derived nanocomposites produces a stable Q-energy state within band-wide energy [5]. Recently, many plants and plant extracts have been used as capping and reducing agent for NP synthesis, improving the field of nanoscience [6]. In addition, plant extracts have additional advantages because they require less time to reduce metal ions. The main reason to consider natural plant extracts to synthesize nanoparticles (NPs) is that it allows selecting environmentally friendly reduction agents, solvent media, and non-toxic substances to stabilize NPs. Ingestion of fruit and its products not only improves the health of individuals, but also reduces the risk of various diseases such as age-related macular degeneration, aging, cardiovascular disease, cancer, eye cataracts, impaired immune system, high blood pressure, high cholesterol, and low-density lipoprotein (LDL) reduction[7,8]. Carotenoids are plant pigments that provide red, yellow, and orange colour to fruits. Approximately 600 carotenoids have been identified, of which approximately 50 are converted to vitamin A. Furthermore, flavonoids are a group of phenol compounds, including anthocyanine, flavanone, catechin, flavanols, flavonoids and isoflavones. To date, about 4000 flavonoids have been identified, most of which are found in citrus and fruit [9], while fruit extracts contain a large amount of reduction agents. For example, blueberries, blackberries, grapes, Terminalia arjuna and Punica granatum L contain a large number of anthocyanides, ascorbic acids, phenolic compounds, flavonoids, sugars and other vitamins [10]. NPs have the potential to show antimicrobial activity as NPs pass through the bacteria's membranes and influence cell activity and metabolic pathways [11]. In addition, ZnONPs derived from extracts of Citrus maxima have been recorded to have significant antimicrobial activity against pathogenic microbes such as Klebsiella aerogenes and S. aureus, and less for E. coli [12]. Nature has its own way of producing highly efficient miniature functionalized materials. The increased responsiveness towards green chemistry and its use in the synthesis of metal nanoparticles led to an aspiration to develop environmentally friendly methods. The advantages of using fruit extracts for the synthesis of nanoparticles are low-cost, economical, energy-efficient, safe and environmentally-friendly, without affecting human health and reducing waste. In this present study, lemon juice extract was used to synthesize Zinc oxide -Titanium di oxide nanocomposite (Zn-TiO2 NC) and characterized using UV-Visible spectrophotometer. The biomedical applications such as antibacterial activity, antioxidant activity, anti-inflammatory activity were tested for the green synthesized nanocomposite.

## Material and Methods:

## Extraction of lemon juice extract:

Fresh lemons were brought from a local supermarket near Poonamallee. The fresh lemons were cut into two pieces and squeezed to get up to 50 mL extract. The freshly collected lemon juice extract was used as a reducing and stabilizing agent to synthesize both zinc oxide and titanium di oxide nanoparticles.

## Green synthesis of Lemon juice mediated titanium dioxide nanoparticles:

0.395g of Titanium oxide was measured and dissolved in 25 mL distilled water. To that, 25 mL filtered Lemon juice extract was added. 20mM of Zinc sulphate was measured and dissolved in 25 mL distilled water. To that, 25 mL filtered Lemon juice extract was added. Then both the reaction mixture was kept on a magnetic stirrer at 600 rpm for 48 h. Meanwhile, UV-Visible spectroscopy was taken to detect the synthesis of Zinc oxide and titanium dioxide nanoparticles. Then these solutions were mixed together and kept on a magnetic stirrer for 700 rpm for 24 h. After that, centrifugation was carried out at 8000 rpm for 10 minutes to separate both the supernatant and the pellet. The supernatant was discarded and the pellet was stored in an airtight Eppendorf tube for further use in characterization and biomedical applications.

## Characterization:

The green synthesized Zinc-titanium di oxide nanoparticles were characterized by using Double beam spectrophotometer (ESICO) with specific time intervals. The morphological characteristics such as size and shape were determined using Transmission Electron Microscope (TEM).

## Antibacterial activity:

The antibacterial activity of the green synthesized zinc and titanium nanocomposite was tested by adopting agar well diffusion technique. Sanguis Mutans Agar was used for this activity. Sanguis mutans agar was sterilized using an autoclave for 15 minutes at 121°C. The sterilized medium was poured on the sterile Petri plates and allowed for the solidification process. A sterile 9 mm polystyrene tip was used to cut well on the surface of agar well plates. The test pathogen Streptococcus mutans, Streptococcus mitis, were swabbed on the agar surface. The Lemon juice mediated Zinc nanocomposite and Titanium dioxide nanocomposite with different concentrations (50 µL, 100 µL, 150 µL) was loaded and the plates were incubated for 24 hours at 37 ° C. After the incubation time, zones of inhibition were measured.

## Antioxidant activity:

## DPPH method:

DPPH assay was used to test the antioxidant activity of biogenic synthesized zinc oxide and titanium dioxide nanocomposites. Diverse concentrations (10µL, 20µL, 30µL, 40µL, 50µL) of lemon juice extract mediated zinc oxide and titanium dioxide nanocomposites was mixed with 1 mL of 0.1 mM DPPH in methanol and 450 µl of 50 mM Tris HCl buffer (pH 7.4) and incubated for 30 minutes. Later, the reduction in the quantity of DPPH free radicals was assessed dependent on the absorbance at 517 nm. Butylated hydroxy toluene was employed as control. Ascorbic acid was enrolled as a standard group. The percentage of inhibition was determined from the following equation (see PDF)

## Anti-inflammatory activity:

## Albumin denaturation assay:

The anti-inflammatory activity for biosynthesized zinc oxide nanocomposite and titanium dioxide nanocomposite was tested by adopting albumin denaturation assay. 0.05 mL of each nanocomposite of various fixation (10µL,20µL,30µL,40µL, 50µL) was added to 0.45 mL bovine serum albumin(1% aqueous solution) and the pH of the mixture was acclimated to 6.3 utilizing a modest quantity of 1N hydrochloric acid. These samples were incubated at room temperature for 20 min and then heated at 55 °C in a water bath for 30 min. The samples were cooled and the absorbance was estimated spectrophotometrically at 660 nm. Diclofenac Sodium was used as the standard. Dimethyl sulphoxide (DMSO) was utilized as a control.

Percentage of protein denaturation was determined utilizing following equation, (see PDF)

## Result and Discussion:

In the present study, the aqueous solution of lemon extract was utilized as a bioactive green reducing agent for reducing zinc and titanium ions to nanocomposites, due to phytochemical compounds present in the plant extracts, and the reaction process was monitored by UV-Visible spectroscopy analysis.

The biosynthesis of Zinc-titanium dioxide nanocomposite using lemon extract showed changes of colour in the aqueous solution from pale white to pale yellow, which was detected using a UV-Vis spectrophotometer. The peak displayed at 380 nm represents Surface plasmon resonance (SPR) which confirms the reducing and stabilizing ability of lemon extract. Previously, Kumar et al. 2020 synthesized hybrid nanocomposite containing Zinc oxide nanoparticles and reported its maximum absorption peak at 374 nm which directly correlates with the UV-Visible results of the current synthesized zinc-titanium nanocomposite [13].

**Figure 1:**
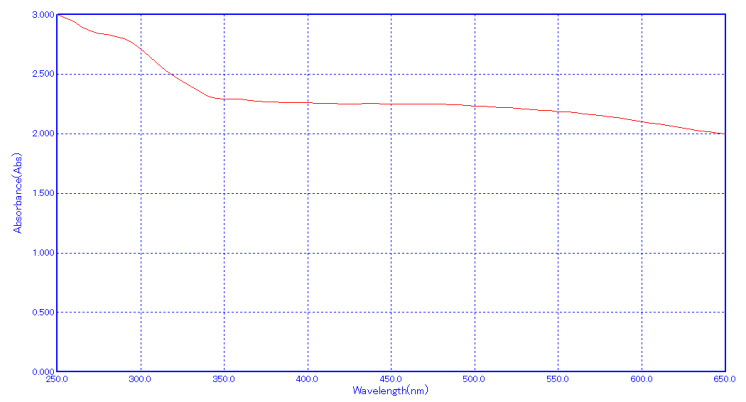
UV-Visible spectra of lemon extract mediated zinc oxide and titanium di oxide nanocomposite.

**Figure 2:**
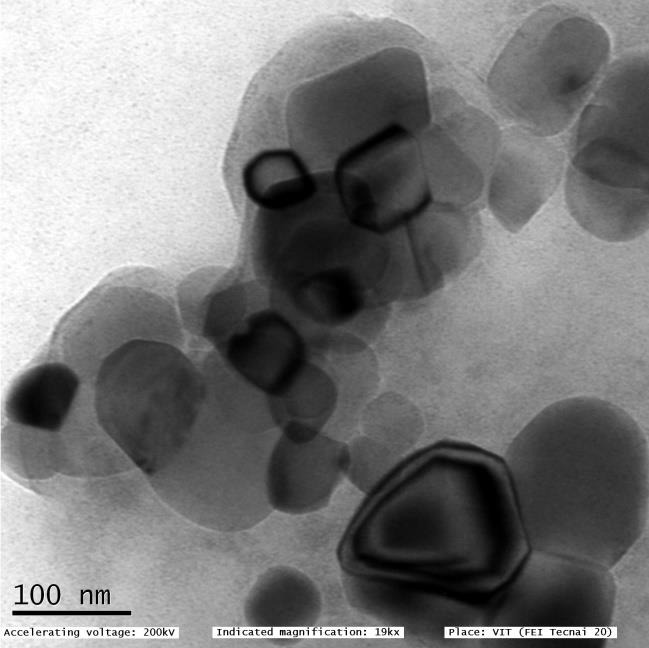
TEM image of green synthesized zinc-titanium nanocomposite

The size and shape of the lemon juice mediated zinc-titanium nanocomposite was characterized by using Transmission Electron Microscope. Figure 1 depicts the TEM image with accelerating voltage 200 Kv and magnification around 19Kx. The TEM image demonstrate that the prepared Zinc oxide -titanium dioxide nanocomposite have a spherical shape with a mean particle size about 25 nm and the intercalation of both zinc oxide and titanium dioxide nanoparticles within chitosan nanoparticles can be clearly seen in Figure 2. In previous research work, Alswat et al. 2016 synthesized Zeolite- zinc oxide nanocomposite which showed mean particle size of about 4 nm with spherical shape [14]. Recently, Elderdery et al.2022 reported the encapsulation of both zinc oxide and Titanium dioxide, with phytocompounds and biopolymer chitosan, which is in accordance with the current TEM analysis [15].

**Figure 3:**
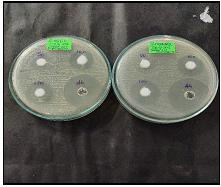
Antibacterial activity of Green synthesized Zinc oxide-Titanium dioxide Nanocomposite (Zn-TiO2 NC)

**Figure 5:**
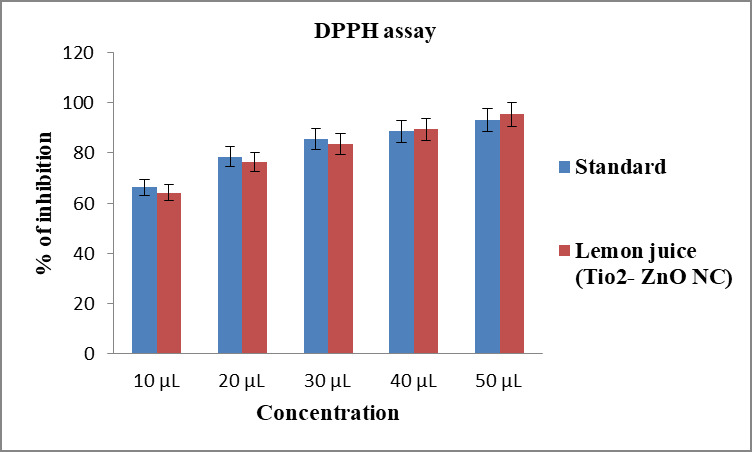
Antioxidant activity of biosynthesized zinc - titanium nanocomposite

The results obtained from the antibacterial activity by well diffusion method revealed that (Zn-TiO2 NC) possess excellent potential against Streptococcus mitis, Streptococcus mutans at increased concentration which was depicted in Figure 3,Figure 5,Figure 5. The bactericidal activity of TiO2 NPs was directly related to concentration dependence. The lemon juice mediated Zinc oxide-titanium dioxide nanocomposite antibacterial activity was determined using the diameter of the inhibition zone. Since, Zn-TiO2 NC showed effective antibacterial activity at 150 µL concentration against S. mitis (28 ± 1.0 mm) followed by S. mutans (25 ± 0.6 mm) and these were more or less equal to bactericidal activity of 150 µL concentration of amoxyrite (34 ± 1.4 mm ). This result suggests that the green synthesized Zn-TiO2 NC effectively act against oral pathogens such as S. mitis and S. mutans. Most of the recent studies have described at least one of the mechanisms for preventing cell wall/membrane synthesis, disrupting energy transmission, producing toxic ROS, photocatalytics, inhibiting enzymes, and reducing DNA production [16].

In previous research works, the antibacterial activity of zinc-titanium dioxide nanocomposite was evaluated. Junejo *et al.* 2021 reported that Zinc-titanium nanocomposite was effective against Escherichia coli at 30mg/ mL concentration [17]. In another recent study, Zinc titanium nanoparticles (ZnTiO3) was synthesized through sol gel process. The antimicrobial activity was studied against Bacillus subtilis and Acinetobacter baumanni and high antimicrobial activity was recorded at 0.4 mg/L concentration for both the pathogens [18]. Therefore, the results obtained from the study revealed the potential antibacterial effect of Zinc-titanium nanocomposite to act against oral pathogens.

## Antioxidant activity:

The antioxidant activity of the synthesized nanocomposite was tested by DPPH assay. The assay was performed with five different concentrations (10-50µL) to identify the potential scavenging effect of the green synthesized Zn-TiO2 NC against oxidative stress. Ascorbic acid was employed as a standard drug and compared with the test sample with same five different concentrations. At initial 10 µL concentration, the nanocomposite showed 64 % scavenging activity whereas standard showed 70 % inhibition. The higher concentration 40 µL of the test sample showed 85 % scavenging activity than the standard drug (83 %). Likewise, at 50 µL concentration, the lemon juice mediated nanocomposite showed higher antioxidant activity upto 94 % whereas standard showed 91 %. This antioxidant activity results significantly implies the use of the novel nanocomposite as free radical scavenger in future dental applications. The antioxidant activity of the zinc-titanium nanocomposite has also been reported in previous and current research works. Previously, El -Borady et al. 2020 evaluated the antioxidant activity of zinc oxide nanoparticles conjugated with biological surface active substance, Folic acid. The DPPH assay results confirmed the higher antioxidant nature of the conjugated zinc oxide nanoparticles at 300 µg/mL concentration with 70 % scavenging potential whereas the standard Vitamin C reported to attain 88 % scavenging activity at the same concentration [19]. Recently, Singh *et al.* 2021 studied the antioxidant potential of amino functionalized polymethacrylate titanium dioxide nanocomposites. The antioxidant activity results of different polymer nanocomposites showed average scavenging activity up to 60.83 % [20]. Therefore, the comparison with previous research works paved a way to understand the higher free radical scavenging potential of the biosynthesized Zinc titanium nanocomposite.

## Anti-inflammatory activity:

**Figure 6:**
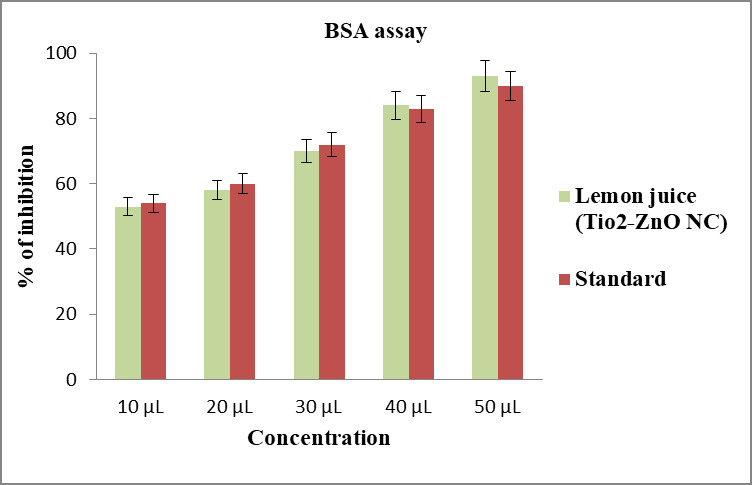
Bovine serum albumin denaturation assay of green synthesized Zinc oxide- Titanium di oxide nanocomposite

Albumin is a globular protein that plays an important role in maintaining plasma pressure and nutritional balance. Various compounds are transported by binding to blood albumin. In addition, human health is closely related to serum albumin concentrations in blood plasma or other biological fluids. Because of its structural similarity to human serum albumin (HSA), bovine serum albumin (BSA) is widely used as a model protein [21]. Hence, BSA is used as a model protein in the current study to identify the anti-inflammatory activity of the biosynthesized nanocomposite. Different concentrations (10- 50µL) of the synthesized Zn-TiO2 NC were used in this study. At minimum concentration 10µL, the nanocomposite revealed its anti-inflammatory activity upto 55 % whereas standard Diclofenac sodium showed 58 %. At 40µL concentration, the lemon juice mediated Zn-TiO2 NC showed 83 % anti-inflammatory activity than standard which showed only 80 %. Similarly, at maximum 50 µL concentration the standard showed its percentage of inhibition up to 86% whereas the green synthesized nanocomposite was 90 %. In Figure 6 previous research work, Pragathiswaran et al. 2020 decorated gold nanoparticles with TiO2@ZnO nanocomposites and evaluated its anti-inflammatory activity using Egg albumin denaturation assay. The anti-inflammatory activity of the nanocomposites was found to be 73 % at 100 µg/mL concentration [22]. Recently, Kamal et al synthesized Biochar-Zinc oxide nano-composite using Zea mays L and the Bovine serum albumin denaturation assay results showed 76 % inhibition at 1000 µg/ mL concentration [23] which further confirms the anti-inflammatory activity of the biosynthesized Zn-TiO2 nanocomposites.

## Conclusion:

Lemon extract is very rich in bioactive molecules, including citric acid, ascorbic acid, minerals, and flavonoids. For this purpose, we used a green, nontoxic, and simple technique for the biosynthesis of zinc oxide -titanium nanocomposite by lemon extract and optimized the different experimental factors including metal ion concentration. The phytochemicals present in the extract may possibly be responsible for the formation of nanocomposite. According to the results of this study, the green synthesized nanocomposite showed a very interesting ability to reduce oral pathogenic bacteria, which highlights the therapeutic value of this nano-composite as antibacterial, antioxidant, anti-inflammatory agent and for use in dental applications.

## Figures and Tables

**Figure 4:**
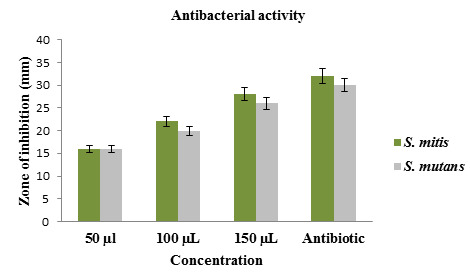
Histogram of Antibacterial activity of green synthesized nanocomposite
